# Ceftriaxone-Associated Severe Acute Hepatitis

**DOI:** 10.7759/cureus.36341

**Published:** 2023-03-18

**Authors:** Muhammad Asif, Wahab J Khan, Sadia Aslam, Ifrah Nadeem, Ashwani K Singal

**Affiliations:** 1 Internal Medicine, Avera McKennan Hospital and University Health Center, Sioux Falls, USA; 2 Internal Medicine, University of South Dakota Sanford School of Medicine, Sioux Falls, USA; 3 Gastroenterology and Hepatology, Avera McKennan Hospital and University Health Center, Sioux Falls, USA

**Keywords:** elevated liver-associated enzymes, transaminitis, drug-induced liver injury (dili), ceftriaxone adverse effects, hepatitis

## Abstract

Drug-induced liver injury (DILI) is a common entity. Ceftriaxone is a well-tolerated parenteral antibiotic widely used for various bacterial infections. We report a patient who developed severe acute hepatitis following a single dose of 2 g ceftriaxone within one day. Apart from a fever of 101.9 F, no other insult was noted to explain his severe hepatocellular injury around the time of presentation. On stopping further ceftriaxone, his symptoms resolved, and liver enzymes normalized within a week. His Roussel Uclaf Causality Assessment Method (RUCAM) score was 6 which suggested DILI be a probable cause of his acute hepatitis. Further surveillance at a larger scale is needed to support evidence for this rare side effect.

## Introduction

Ceftriaxone, a third-generation cephalosporin, is a commonly used broad-spectrum antibiotic for several bacterial infections affecting various organ systems, including skin and soft tissue. Significant side effects include hypersensitive reaction, skin rash, hemolytic anemia [1], and C. difficile colitis. Cholestasis and hepatitis are known side effects of this drug. However, they are primarily mild, occur days to weeks after the drug initiation [2], and are self-limited in the majority [3]. We describe a case of a healthy male who developed severe acute hepatitis following a single dose of intravenous ceftriaxone that he received for a suspected tooth infection.

## Case presentation

A 50-year-old male with a pertinent medical history of type 2 diabetes mellitus, situs inversus, and obesity was seen by his dentist for one week of toothache. He was diagnosed to have maxillary tooth decay and a possible infection. As the tip of this tooth was not amenable to restoration, the patient underwent dental extraction. Due to continued pain around the extraction site, one episode of non-bloody nonbilious vomiting, chills and subjective fevers, he presented to the emergency room on the second day after the extraction. He was noted to have a fever of 101.9 that resolved during his stay in the emergency room with one dose of ketorolac. His other vital signs were within normal limits (wnl). He was given 2 g of ceftriaxone injection and was advised to start Augmentin the next day. The patient was discharged home and within 12 hours started experiencing bloatedness, fatigue and significantly dark-colored urine. Unable to start Augmentin, he re-presented the clinic. He denied abdominal pain, any further fever, chills, dizziness, vomiting or diarrhea. His vital signs were wnl. General physical and abdominal examinations were negative for scleral icterus, altered mental status, or abdominal tenderness. No rash or lymphadenopathy was noted.

The initial workup showed alanine transaminase (ALT) over 5,000 U/L (Ref: 4-33 U/L), aspartate aminotransferase (AST) of over 10,000 U/L (Ref: 13-39U//L), and alkaline phosphatase (ALP) of 159 U/L (Ref: 34-104 U/L). He had elevated bilirubin in urine. Serum bilirubin, lactic acid, ammonia, albumin, and INR were normal. Complete blood count was notable for low white cell count at 3.7 K/UL (Ref: 4.5-11 K/UL) with elevated eosinophil count at 11% (Ref: <7%). Subsequently, he was hospitalized with a diagnosis of acute hepatocellular injury. The patient denied using any alcohol, recreational drugs, or any other over-the-counter or herbal products including Tylenol. There was no previous diagnosis of liver disease. The patient had managed diabetes mellitus with oral hypoglycemic agents, including sitagliptin (Januvia), empagliflozin (Jardiance), and metformin.

**Figure 1:**
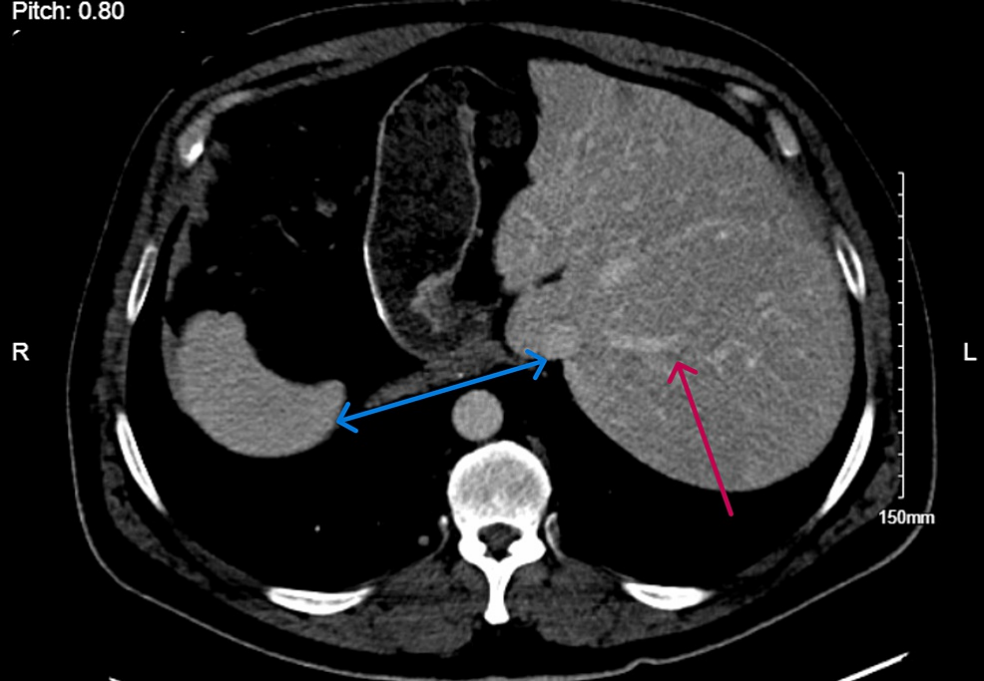
CT abdomen with contrast showing situs inversus (blue double-headed arrow), fatty liver and no focal hepatic lesion (red arrow)

Comprehensive serological workup was negative for toxicology (alcohol, acetaminophen, salicylate); viral panel (IgM to hepatitis A and B viruses, HBsAg, and antibodies to hepatitis C or human immunodeficiency viruses, COVID-19, cytomegalovirus (CMV), epstein-barr virus (EBV), herpes simplex virus (HSV) and influenza); iron profile (saturation and serum ferritin), and autoimmune panel (immunoglobulin G level and smooth muscle antibody) were unremarkable. CT scan of the abdomen and pelvis with IV contrast showed previously known situs inversus totalis, surgically absent gallbladder, and mild fatty infiltration of the liver. There was no evidence of cirrhosis, biliary dilatation, lymphadenopathy, or focal liver lesion (Figure 1).

A diagnosis of ceftriaxone-induced acute hepatitis was made, and the patient was treated conservatively by holding any further ceftriaxone. The liver enzymes started improving within the next 24 hours, with AST to 109 U/L and ALT to 323 U/L. The patient remained asymptomatic with normalization of urine color and was discharged home without any new medications or antibiotics. On follow-up after 10 days, the liver enzymes completely normalized, and eosinophilia also resolved.

## Discussion

Drug-induced liver disease may account for 10% to 50% of adult patients with elevated enzymes, especially in patients over age 50 years [4]. Based on laboratory values, liver injury is classified into hepatocellular, cholestatic, or mixed. Drug-induced liver injury (DILI) mechanisms include direct cellular toxicity, idiosyncratic reaction, and immune-mediated injury [5]. Before determining a diagnosis of DILI, it is crucial to rule out other competing etiologies of acute hepatitis. Other than toxic hepatic injury, ischemic hepatitis is another etiology where significantly elevated liver enzymes can be seen from any mechanism leading to under-perfusion of the liver. In our patient, there were no hypotensive or hypoxic events noted. He presented to ER immediately after having a fever and one episode of vomiting. His heart rate was normal and blood pressure was on the higher side of normal even at the peak of his illness. He never required any supplemental oxygen. His history and physical exams were not indicative of evidence of significant dehydration. Another hallmark of DILI is the improvement in liver biochemical tests on the secession of the suspected culprit medication, as it happened in our patient.

In DILI evaluation, Roussel Uclaf Causality Assessment Method (RUCAM) is an objective means of assigning points for clinical, biochemical, serologic, and radiologic features of liver injury, giving an overall assessment score that reflects the likelihood that the hepatic injury is due to a specific medication. The criteria-based score calculated for our case is summarized below (Table 1).

**Table 1:** RUCAM Score calculated for the patient

Checked Variables	Score
Time of onset	1
Course	3
Risk factors	0
Concomitant drugs	0
Search for nondrug causes	0
Previous information on hepatotoxicity	2
Response to re-administration	0
Total score	6

The calculated RUCAM score was 6, which suggests DILI to be the probable cause of our patient's hepatocellular injury [6]. This case interestingly demonstrates a rare occurrence of ceftriaxone-associated acute hepatocellular severe injury. Ceftriaxone inhibits bacterial cell wall synthesis by binding to the penicillin-binding proteins. It can only be administered parenterally and has good penetration to soft tissue, CNS, abdomen, and bones. In adults with normal renal and hepatic function, it has a half-life of 5 to 9 hours and is excreted via bile and urine. Elevated liver enzymes and cholestasis are reported in up to 6% of the patients however, most of the time, it is mild, and the medication can be continued with close monitoring of liver function tests. However, it has rarely been reported to cause severe DILI, less than 1%, and acute hepatitis [7-9], necessitating termination of further treatment. It is tough to predict which patient will have a severe liver injury. However, close monitoring of liver function can help identify those patients who will benefit from changing the antibiotic regimen for safety issues.

The treatment of DILI is mainly the withdrawal of the offending agent and supportive care with monitoring of liver functions. Acetylcysteine has been used in liver injury caused by acetaminophen, and L-carnitine has been used in cases of valproic overdose. Steroid use has been found to be effective in autoimmune or severe idiosyncratic reaction-related liver injury that has happened with DRESS syndrome (drug reaction with eosinophilia and systemic symptoms). Liver biopsy can be performed, however, was not done in our patient due to rapid improvement during the hospital course and no signs of acute liver failure. If conservative care fails, sometimes people with acute liver failure secondary to DILI need liver transplantation.

## Conclusions

Ceftriaxone is a widely used broad-spectrum antibiotic medication. Severe acute hepatitis is a rarely reported side effect of this drug. By reporting this case, we intend to spread awareness about this entity so that clinicians can use this drug safely and stop immediately if such a complication is seen in their patients after ruling out any other competing etiology. The limitation of this case report is the possible contribution of tooth infection-related stress. Therefore, it is hard to establish the causation effect with full certainty. Further studies and data are needed to support or refute this association.
